# Revealing posturographic profile of patients with Parkinsonian syndromes through a novel hypothesis testing framework based on machine learning

**DOI:** 10.1371/journal.pone.0246790

**Published:** 2021-02-25

**Authors:** Ioannis Bargiotas, Argyris Kalogeratos, Myrto Limnios, Pierre-Paul Vidal, Damien Ricard, Nicolas Vayatis

**Affiliations:** 1 Centre Borelli CNRS INSERM, ENS Paris-Saclay, Paris-Saclay University, Gif-sur-Yvette, France; 2 Centre Borelli CNRS INSERM, Université de Paris, Paris, France; 3 School of Automation, Hangzhou Dianzi University, Zhejiang, China; 4 Neurology Department, HIA Percy, Service de Santé des Armées, Clamart, France; São Paulo State University (UNESP), BRAZIL

## Abstract

Falling in Parkinsonian syndromes (PS) is associated with postural instability and consists a common cause of disability among PS patients. Current posturographic practices record the body’s center-of-pressure displacement (statokinesigram) while the patient stands on a force platform. Statokinesigrams, after appropriate processing, can offer numerous posturographic features. This fact, although beneficial, challenges the efforts for valid statistics via standard univariate approaches. In this work, 123 PS patients were classified into fallers (PS_F_) or non-faller (PS_NF_) based on the clinical assessment, and underwent simple Romberg Test (eyes open/eyes closed). We developed a non-parametric multivariate two-sample test (ts-AUC) based on machine learning, in order to examine statokinesigrams’ differences between PS_F_ and PS_NF_. We analyzed posturographic features using both multiple testing with *p*-value adjustment and ts-AUC. While ts-AUC showed significant difference between groups (*p*-value = 0.01), multiple testing did not agree with this result (eyes open). PS_F_ showed significantly increased antero-posterior movements as well as increased posturographic area compared to PS_NF_. Our study highlights the superiority of ts-AUC compared to standard statistical tools in distinguishing PS_F_ and PS_NF_ in multidimensional space. Machine learning-based statistical tests can be seen as a natural extension of classical statistics and should be considered, especially when dealing with multifactorial assessments.

## Introduction

Postural control is the capacity of an individual to maintain a controlled upright position. Falls have been reported as one of the major causes of injury among elderly and more importantly among patients of balance-related disorders, such as Parkinsonian syndromes (PS). It has been estimated that one third of the population over 65 years-old faces minimum one fall per year [1]. Falls promote the decrease in mobility, problems of autonomy in daily activities (bathing, cooking, etc.), or even death [1, 2]. Taking also into consideration the aging of many modern societies, accurate risk assessment has become a major challenge with huge socio-economic impact [3].

Force platforms are one of available acquisition tools of clinical researchers for the assessment of postural control. Such platforms record the displacement of the center of pressure (CoP) applied by the whole body in time while the individual stands upon it and follows the clinician’s instructions. These CoP trajectories, usually called statokinesigrams, have been widely used in assessing the balance disorder in healthy or PS populations. It has been shown that CoP displacement characteristics can reflect individuals’ postural impairment when special acquisition protocols are followed [2, 4, 5].

Clinical research often aims to find the significant differences between fall-prone individuals and others who have not yet manifested important balance impairment. Researchers usually compute several features using signal processing techniques and evaluate their usefulness relying on a variety of available univariate tests, such as the Student’s t-test, Kolmogorov–Smirnov or Mann-Whitney Wilcoxon. However, usually in experimental works, where pre-planned hypotheses are not well-fixed, multiple univariate tests are applied consecutively in order to find the features that separate significantly the two groups. The aforementioned multiple testing scheme has been part of a well-known scientific debate [6], mainly criticized for the increased probability of reporting a false-positive finding. More specifically, it has been reported that for alpha level *α* = 0.05, it is possible that 1 in 20 relationships may be statistically significant but not clinically meaningful [6]. Thus, several biostatisticians recommend to disclose all the elements of the conducted analysis, and not only the elements that found to be significant. The violation of this recommendation and the regular misuse of those tests [7] combined with the relatively small available cohorts, may lead to false conclusions and as a consequence to a significant lack of clinical consensus or at least delay in reaching it. Well-known adjustments have been proposed in order to limit the aforementioned probability of a false-positive finding (such as Bonferroni corrections) but they have been reported as conservative compromises (due to the significant increase of the probability for false-negative output) [6] that do not constitute a satisfactory solution [8]. Other corrections (more powerful than Bonferroni) such as Hommel [9], Hochberg [10] and Holm [11] (in descending power order [12]) have been also proposed.

Classic statistical tests are very sensitive on the size of the available dataset. The generalization of any result is not safe when only relatively small populations are available (see [13] for the high risk of making false conclusions). In order to reduce this sensitivity, machine learning algorithms assess their results using cross-validation schemes. Briefly, an algorithm trains a model that ‘learns’ to solve the problem in a randomly selected part of the dataset (called training-set), and then tests whether it can be effective on the rest of the ‘unseen’ data (test-set). The learning and validation process is repeated multiple times and performance metrics are averaged. In the context of multidimensional datasets with binary labels {−1, +1}, the idea of assessing the separability of two groups is based on the aforementioned learning and validation scheme. The learning process sets the criteria in order to rank the population in the test-set by means of a scoring function *s*. Those who are ranked at the top of the list will be considered to belong to the positive class [14]. The machine learning community has recently made significant progress in this topic [15–18], especially related to the design of appropriate criteria for the characterization of the ranking performance and/or meaningful extensions of the Empirical Risk Minimization (ERM) approach to this framework [19, 20]. In a large part of these efforts, the well-known criterion of the area under the ROC curve (AUC) is considered as the gold standard for measuring the capacity of a scoring function to discriminate groups of populations [14]. Briefly, in the setting of two-sample statistical testing, an algorithm ‘learns’ the rule that maximizes the AUC between the two groups in the training-set, and then tests the applicability of this rule to the test-set during the validation process.

Unfortunately, to the best of our knowledge, these novel advancements in statistical testing remain largely unexploited by the parkinsonism-related community. The lack of common language and proper methodological simplifications to make the approaches easy to understand by clinical researchers are possibly the major reasons for such an observed distance.

In postural research, simple acquisition protocols (such as the basic Romberg test) have been reported to contain inconclusive information to evaluate sufficiently the postural control of an individual [21]. However, only recently, works proposed that a combination of multiple global features, derived from CoP trajectories using data mining techniques, might be advantageous in order to classify fallers and non-fallers. Earlier works [22, 23], showed that although none of the features alone could classify effectively elderly fallers/non-fallers (i.e. weak classifiers), yet combining all features through non-linear multi-dimensional classification gave significant results. It is suggested that the shape of the decision surface lies indeed in a multidimensional space and should be learned using multiple features at once. As a consequence, the above findings raise reasonable questions about the ability of traditional statistical tools and testing protocols to fully reveal and exploit the existing associations.

The objective of the present study is to propose an easy-to-use and -interpret two-sample hypothesis testing approach, in an attempt to address some the aforementioned difficulties of clinical research. Our contribution is to propose a new variation of a multivariate two-sample test through AUC maximization, which was originally theoretically established in [14], and test it to a PS population which includes two groups: fallers (PS_F_) and non-fallers (PS_NF_). We intend to highlight the benefits that one might have by using such kind of two-sample analysis in the presence of multiple features, and demonstrate the contradicting conclusions that a traditional statistical analysis (hypothetical future clinical study) might have had compared to the proposed method. In addition, we performed comparative performance in simulated synthetic data in order to strengthen the evidence that the proposed approach is statistically sound and consistent. Therefore, we decided to conduct such a study, providing it though in the Appendix in order to keep the main text focused on the problem-specific results in which we are primarily interested.

## Materials and methods

### Balance measurements and fall assessment

**Fig 1:**
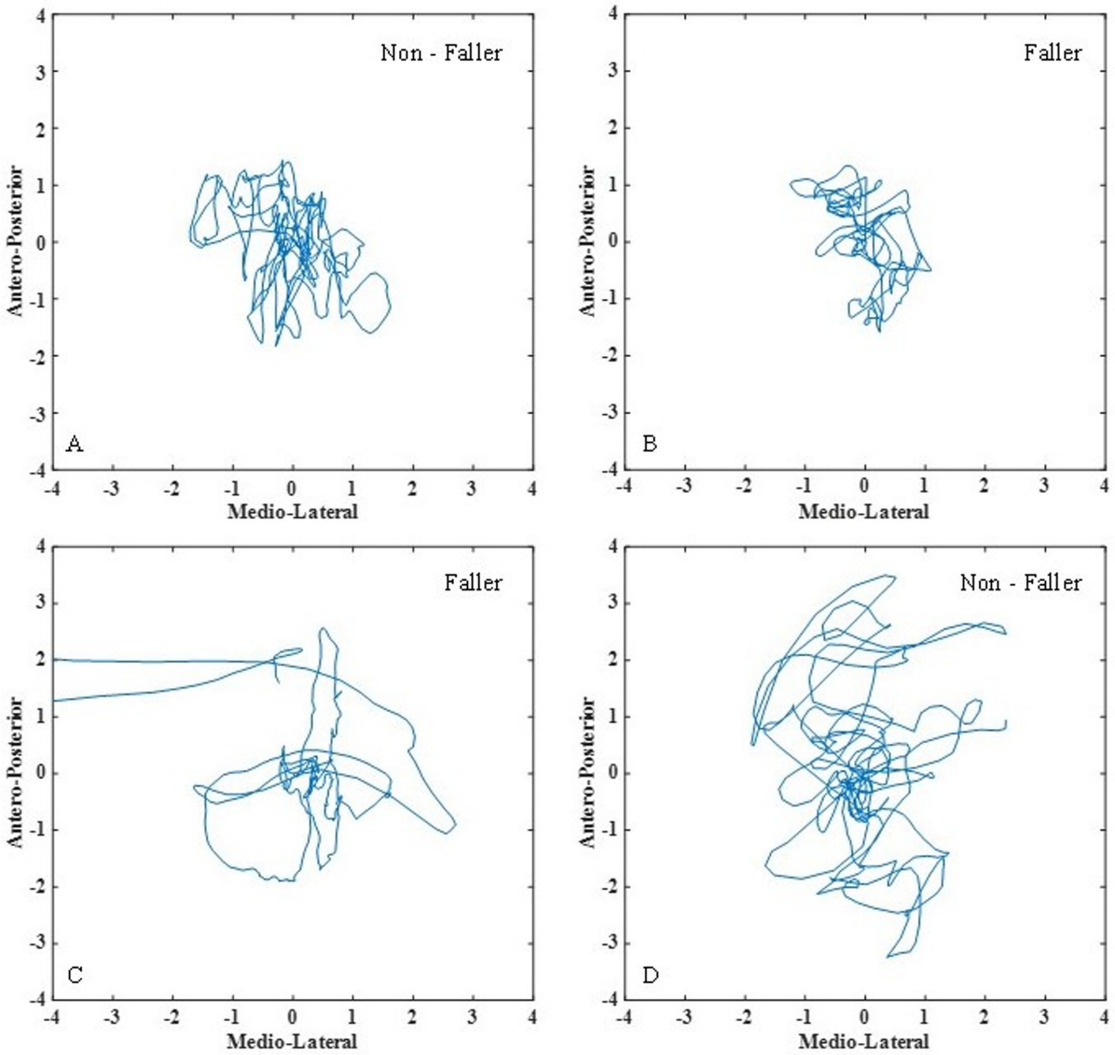
Examples of statokinesigrams from fallers and non-fallers. The x-axis is the medio-lateral (ML) movement and the y-axis is the antero-posterior (AP) movement of the body in centimeters (cm) during the acquisition. As it can be observed, fallers and non-fallers are not easily distinguishable by examining visually their statokinesigrams.

Our dataset comes from the Neurology department of the HIA, Percy hospital (Clamart, France), and includes 123 patients (78.7 ± 5.4 years-old, Table 1) who suffered from Parkinsonian syndromes. PS patients that suffered from other comorbidities (such as vestibular and proprioceptive impairements) were not included in the study. Following the acquisition protocol, patients were asked to remove their shoes and to maintain upright position on a force platform keeping their eyes open and their arms at the side. The CoP trajectory was recorded for 25 seconds at that stance. After that, patients were asked to close their eyes maintaining their upright position. After a ten-second pause, clinical experts recorded 25 additional seconds with eyes closed (Fig 1).

**Table 1:** Population characteristics: The 123 patients included in the study.

Characteristics	Non-Fallers	Fallers
Population size	99	24
Age	78.8 ± 5.3	78.5 ± 5.9
Gender	M:71/W:28	M:16/W:8
UPDRS III total score	23.6 ± 11.9	26.3 ± 11.1
Disease duration	4.7 ± 3.5	5.7 ± 4.2

Statokinesigrams were acquired using a Wii Balance Board (WBB) (Nintendo, Kyoto, Japan), which has been found to be a suitable and convenient tool for the clinical setting [24, 25], and the newly proposed portable package developed in our laboratory. Statokinesigram from WBB are sent to the clinician‘s professional Android tablet via Bluetooth connection. Acquired signals are sent (after anonymization and encryption) to a central database for high level processing (computation of features associated to postural control and application of appropriate algorithms [22, 23, 26]), and the demanded results are communicated to the clinician online. Since the WBB records the CoP trajectories at non-stable time resolution, the acquired statokinesigrams are resampled at 25Hz using the SWARII algorithm [27].

In order to label the participants, a questionnaire (implemented to the Android tablet) was filled for every subject registering information about falls during the last six months prior to the examination. As in previous works [28], participants were labeled as fallers (PS_F_) if they had come to a lower level near the ground unintentionally at least once during that period. Twenty-four (24) patients were labeled as fallers. Any useful information about the conditions of falls were registered. The clinical trial registered at ANSM (ID RCB 2014-A00222-45) was approved by the following ethics committee/institutional review board(s): 1) Ethical Research Committees (CPP), Ile-de-France, Paris VI; 2) French National Agency for the Safety of Medicines and Health Products (ANSM); 3) National Commission on Informatics and Liberty (study complies with the MR-001). All research was performed in accordance with relevant guidelines and regulations. After information and allowing adequate time for consideration, written informed consent was obtained from all participants before being included in the study.

### Choice of posturographic features

Our analysis included only features that were computed on the two-dimensional CoP displacement and have been previously proposed as indicators of postural impairment [2, 29, 30]. Table 2 provides the names, measuring units, and descriptions (where needed) for the features that were included in the test.

**Table 2:** Computed features derived from the CoP displacement during the acquisitions.

Feature	Unit	Description
RangeX	cm	–
MaxX	cm	Maximum medio-lateral displacement (right)
MinX	cm	Minimum medio-lateral displacement (left)
VarianceX	cm^2^	–
VelocityX	cm/s	Average instant x-axis velocity of CoP changes
AccelerationX	cm/s^2^	Average instant x-axis acceleration of CoP changes
F95X	Hz	Frequency below which 95% of the x-axis CoP trajectory’s energy lies
RangeY	cm	–
MaxY	cm	Maximum antero-posterior displacement (front)
MinY	cm	Minimum antero-posterior displacement (back)
VarianceY	cm^2^	–
VelocityY	cm/s	Average instant y-axis velocity of CoP changes
AccelerationY	cm/s^2^	Average instant y-axis acceleration of CoP changes
F95Y	Hz	Frequency below which 95% of the y-axis CoP trajectory’s energy lays)
DistC	cm	Instant distance from the center of the trajectory
EllArea	cm^2^	Confidence ellipse area that covers the 95% of the trajectory’s points
AngularDeviation	degrees	Average of the angle of deviation

### Two-sample test through AUC optimization (ts-AUC)

**Fig 2:**
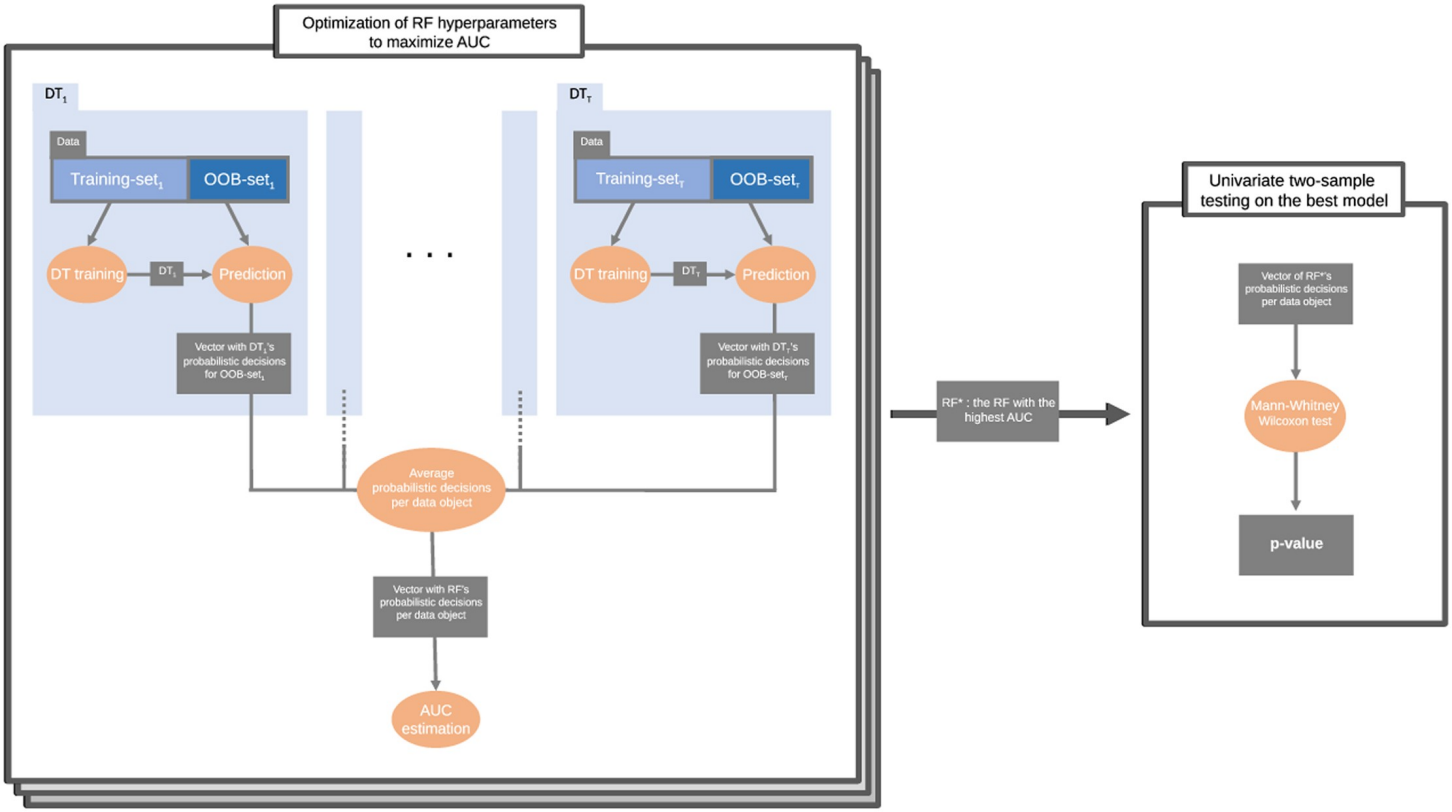
Scheme of the ts-AUC algorithm. In order to find the AUC* (maximal AUC), a number of Random Forests (RFs). For the RF* with the best AUC*, the univariate Mann-Whitney Wilcoxon non-parametric two-sample test is applied on the average posterior probability values of the whole population.

We applied a bootstrap aggregation classification, in particular a random forest (RF) [31] that comprises several decision trees (DTs). Therefore, in the development of each DT, only a part of the whole dataset does participate (in-bag) while the other part is left out (out-of-bag, or OOB). Consequently, the OOB subset can be used as test-set for the particular DT. In our approach, instead of the originally proposed testing method based on data splitting, we used the predictions of the OOB population [32]. The number of DTs was large enough (*T* = 200) compared to the actual population. The individuals can be selected in different OOB sets more than once. Every time an individual is part of an OOB set, the corresponding DT outputs the probability for him/her being a PS_F_ or a PS_NF_. This is computed as the fraction of individuals of the positive class (fallers) in the tree leaf where he/she reaches. Thus, his/her final score is given by the average of the posterior probabilities over the trees he/she was part of the OOB set (see Fig 2). Averaged posterior probabilities (*P*) of the positive class (fallers) are used in order to compute the Mann-Whitney *U*-test statistic, denoted by *U* as proposed in the theoretical work of [14]. The empirical AUC for the chosen hyperparameters is given by $\frac{U}{N_{\text{F}}\cdot N_{\text{NF}}}$. Briefly, the null hypothesis, H_0_, and the alternative one, H_1_, are expressed as follows:

$$\begin{array}{c} “\;{\text{H}}_{\text{0}}:\;{\text{AUC}}^{*}=\frac{1}{2}\;”\;vs.\;“\;{\text{H}}_{\text{1}}:\;{\text{AUC}}^{*}>\frac{1}{2}\;”. \end{array}$$ (1)

The OOB percentage was fixed to 36.8% of the included population. Searching the empirical AUC* (maximal AUC), the hyperparameters that are optimized are the leaf-size *LS* and the number of features to be used to build each tree *M*. We avoided a greedy approach using a Bayesian optimization process, where only relatively shallow (7 < *LS* < 20) and simple (*M* < 9) DTs were allowed to be tested. The averaged posterior probabilities of the *star model*, where AUC = AUC*, are used to compute the scoring function (and the *p*-value) through a univariate Mann-Whitney Wilcoxon (MWW from now on) test on the whole available dataset (see Algorithm 1 and Fig 2).

**Algorithm 1** The proposed ts-AUC statistical test.

**Input**: *X* and *Y* are the points’ coordinates of the trajectory (statokinesigram); Leaf-size(*LS*), Out-of-bag(*OOB*) and Nnmber of features(*M*) are vectors with the required hyperparameters.

**Output**: AUC*, RF*, P*, *p*-value*.

 ***Step 1*: *Exploration of the space of hyperparameters***

1: **for** *i* ∈ *LS* **do**

2:  **for** *j* ∈ *M* **do**

3:   *RF* = RandForest(*X*, *Y*, *LS*_*i*_, *M*_*j*_)

4:   *P* = OOBpredict(*RF*_*i*,*j*_)

5:   *U* = Mann_Whitney_Utest_Statistic(*P*)

6:   *AUC*_*i*,*j*_ = AUCestimation(*U*, *Y*)

7:  **end for**

8: **end for**

 ***Step 2*: *Choose the best model and apply MWW***

9: (i*, j*) = argmax_*i*∈*LS*,*j*∈*M*_ *AUC*_*i*,*j*_

10: AUC* = *AUC*_i*,j*_

11: RF* = RandForest(*X*, *Y*, *LS*_i*_, *M*_j*_)

12: P* = OOBpredict(RF*)

13: *p*-value* = MWW(P*, *Y*)

### Out-of-bag feature importance

Additionally, the proposed algorithmic modifications allow the assessment of the importance of each feature to the ts-AUC’s final decision. We estimated the out-of-bag feature importance by permutation. Briefly, the more important a feature is, the higher its influence (i.e. the increase) would be to the model’s error after feature’s random permutation at the OOB subset. The permutation of a non-influential feature will have minimum, or no effect at all, on the model’s error. Having *D* features in the dataset and *T* trees in the RF model, the influence of feature *j* ∈ {1, …, *D*} is computed as:

$$\begin{array}{c} I_{j}=\frac{\;d_{j}\;}{\;\sigma_{j}\;}, \end{array}$$ (2)

where *d*_*j*_ is the average change of model error after the permutation of feature *j*, and *σ*_*j*_ is the standard deviation of the above change. Important to explain that every feature *j* participates only to the training of a subset of the trees of the RF. Therefore, *d*_*j*_ and *σ*_*j*_ are derived by those trees in which the feature *j* was selected to participate in their training.

Since our objective is to enhance interpretability of results, our feature importance analysis aims to identify all the important features, even those which are redundant or colinear, rather than finding a parsimonious set of important features. Hence, we followed the additional procedure proposed in [33] especially for interpretation purposes. Briefly, we computed the AUC of the OOB (AUC_OOB_) of RFs starting from the most important feature, and adding progressively all the others in descending importance order. The best model is the smallest model (less features) with an AUC_OOB_ higher than the maximum AUC_OOB_ reduced by its empirical standard deviation (based on 20 runs).

### Experimental settings

We compare the results obtained by the proposed ts-AUC with the Maximum Mean Discrepancy test (MMD-test) [16], which is a well-established multivariate test and state-of-the-art in terms of performance. The MMD measures the maximum difference between the mean of two data samples, in the space of probability measures of a Reproducing Kernel Hilbert Space (RKHS). Practically, this test uses the unbiased squared MMD statistic. It has been proven to be highly efficient and easy to use (a package with kernel optimization is provided in [34]).

In addition, we compare the results of ts-AUC with standard statistical testing approaches which are usually used in clinical studies. We checked the *p*-values of all 17 features (i.e. *D* = 17) with the labels {‘faller’/‘non-faller’} using the non-parametric Mann-Whitney Wilcoxon test. Typically, clinicians would report those features which were found statistically significant (e.g. with *p*- value < *α* = 0.05) and any interesting non-significant finding.

In order to prevent the increase of the false positive probability due to the large number of tested hypotheses, *p*-value adjustment procedures are applied. We use the Bonferroni correction, which is the most widely used *p*-value adjustment in biomedical research. Moreover, after taking into account the criticism that Bonferroni has received [8], we also apply alternative approaches such as Hommel [9], Hochberg [10], Holm [11] and Bonferroni corrections.

We assess the effect of population size to the final result by performing the following two additional experiments:

1. We progressively decrease, uniformly at random, the population size by a step of 10% (from 95% to 35%).
2. We progressively reduce, uniformly at random, the number of PS_NF_ by a step of 10% (from 95% to 35%).

At every step, the analysis of each case runs 12 times and the percentages of significant results were compared (see Figs 6 and 5).

Finally, to enhance further our conclusions, we compared the behaviour of the tests to simulated groups with various populations (N from 100 to 200), various levels of separation (difference in mean values) and various class proportions between the two groups (50/50, 70/30, 90/10, percentages of positives/negatives). These results can be found in the Appendix (see Figs 7, 8, 9, 10, 11, 12, 13).

## Results

The presented ts-AUC test was applied using the features derived from statokinesigrams from Eyes-Open and Eyes-Closed acquisitions. Table 3 contains the obtained *p*-values for the two groups by the application of the ts-AUC and MMD tests. Both these tests agreed that the features derived by statokinesigrams of Eyes-Open significantly separated PS_F_ from PS_NF_, contrary to those from Eyes-Closed that did not show a significant result (Table 3). Therefore, we will henceforth continue by presenting detailed analysis only for Eyes-Open features.

**Table 3:** The *p*-values obtained by the application of the ts-AUC and MMD tests on the features extracted from Eyes-Open and Eyes-Closed statokinesigrams.

Data type	MMD result	ts-AUC result
Eyes-Open	H_0_ rejected *	*p*- value < 0.01 *
Eyes-Closed	H_0_ not rejected	*p*- value > 0.05

Features derived by Eyes-Closed statokinesigrams did not show a statistically significant result neither using ts-AUC nor MMD test. Therefore the study did not proceed to further analysis of these statokinesigrams. The statistically significant results are indicated by ‘*’.

**Fig 3:**
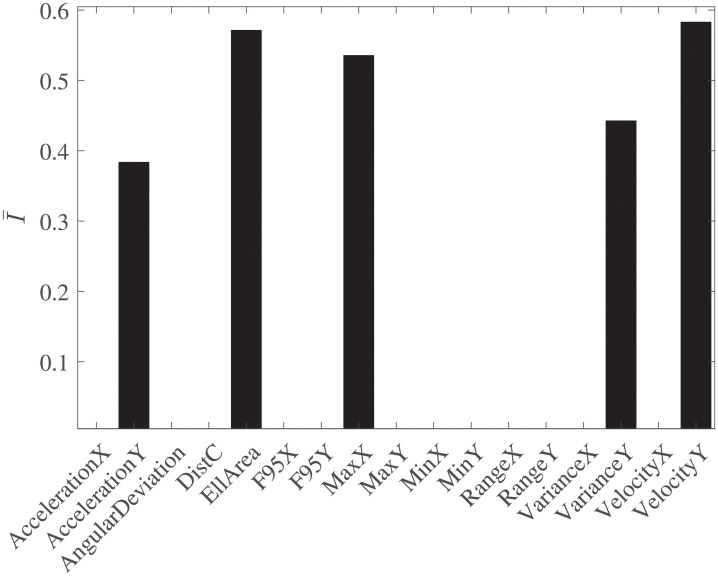
Important features. The importance of features as estimated by applying the approach of [33] using the hyperparameters that produced the RF*.

**Fig 4:**
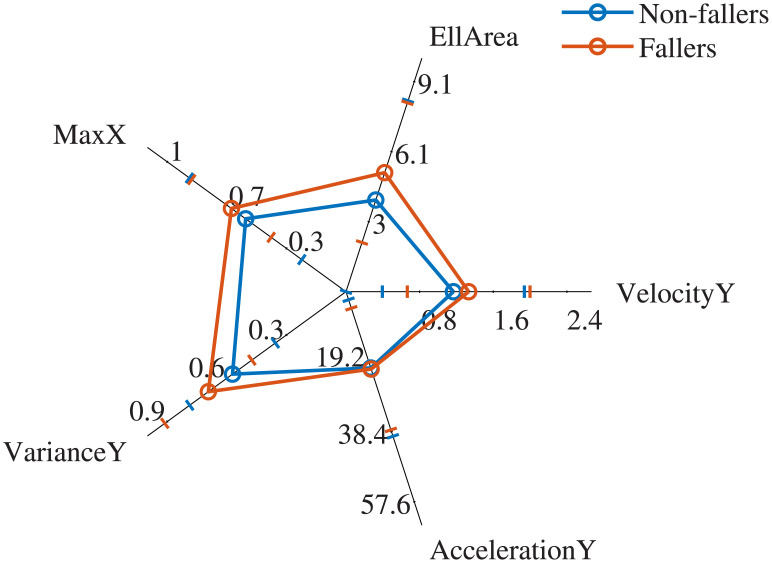
Radar chart. Radar chart comparing fallers and non-fallers based on the mean (o) ± standard deviation (-) of the most important features of our analysis. All six features are positively correlated with low postural control, which justifies the meaningfulness of inspecting the area of the curves in this chart. The profile of the two groups is significantly different.

The most influential features were found to be the VelocityY, VarianceY, AccelerationY, EllArea (Confidence Ellipse area) and MaxX (see in Fig 3 their relative importance and in Fig 4 their mean ± standard deviation per group). Table 4 indicates those features that showed *p*- value < 0.05 and the decisions regarding statistical significance obtained after applying each of the three employed corrections. In every row of Table 4, values at column 1 compared one by one to values at columns 2, 3 and 4 were found **always higher**. Interestingly, although the AccelerationY did not show statistical significance after the MWW application (*p*- value > 0.05), it was found as one of the influential features by the ts-AUC test. According to Table 4, using the results from the three corrections with level *α* = 0.05, none of the features would reject the H_0_ of two-sample MWW test.

**Table 4:** Significant and non-significant results of a univariate two-sample Mann-Whitney Wilcoxon (MWW) test, and the *p*-values after Hommel, Hochberg, Holm and Bonferroni corrections.

	*p*-value before correction	*p*-value after correction			
Feature	*p*-value	Hommel	Hochberg	Holm	Bonferroni
EllArea	0.0045	0.058	0.071	0.071	0.072
VarianceY	0.006	0.092	0.11	0.11	0.12
MaxY	0.006	0.092	0.11	0.11	0.12
DistC	0.007	0.10	0.11	0.11	0.13
RangeY	0.008	0.12	0.13	0.13	0.17
VelocityY	0.009	0.24	0.33	0.36	0.50
MaxX	0.03	0.32	0.33	0.36	0.51
RangeX	0.04	0.34	0.41	0.47	0.79
VarianceX	0.04	0.36	0.41	0.47	0.82
MinY	0.04	0.41	0.41	0.47	0.87
MinX	>0.05	-	-	-	-
VelocityX	≫	-	-	-	-
AccX	≫	-	-	-	-
F95X	≫	-	-	-	-
AccY	≫	-	-	-	-
F95Y	≫	-	-	-	-
AngularDev	≫	-	-	-	-

After all corrections, none of the *p*-values were found lower than *α* level of 0.05. Therefore, none of the features can safely reject the null hypothesis at the default 5% significance level.

### Population size

**Fig 5:**
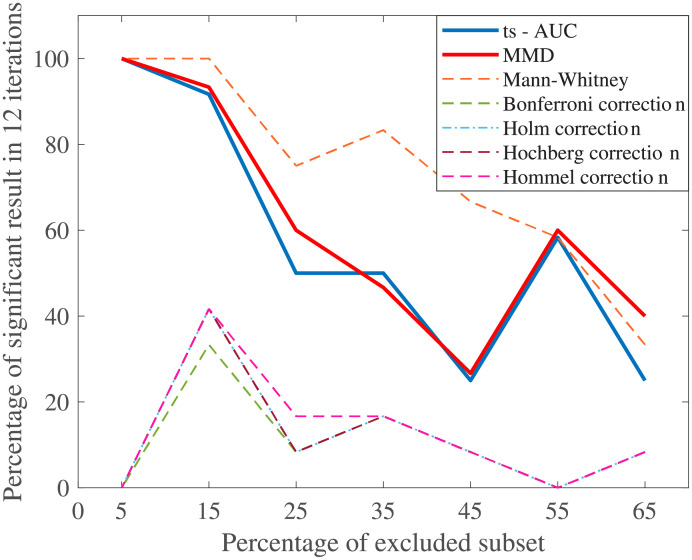
The average performance of two-sample testing approaches with smaller population. The dataset size was progressively decreased by a step of 10%. The included subset of each step was selected uniformly at random 12 times and the tests run in every iteration. We observe that ts-AUC and MMD have almost the same performance. Decreasing the population leads to lower chance of distinguishing the two groups. On the other hand, all the two-sample corrections present significantly lower performance.

As expected, the decrease of population size had an important effect to the performance of all tests. Both ts-AUC and MMD test showed similar behavior with the progressive decrease of population size. Specifically, the number of times that the fallers and non-fallers were found statistically different was gradually decreased. After 55% of population size decrease, the two groups were found significantly different in less than 50% of the cases (Fig 5). Univariate testing through MWW followed a similar decrease. Multiple testing showed that the groups cannot be considered as statistically different(almost always).

**Fig 6:**
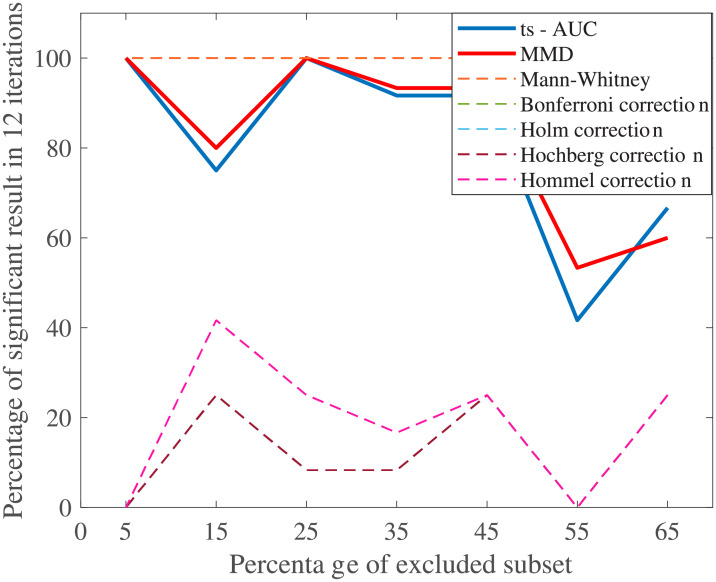
The average performance of two-sample testing approaches with smaller non-faller population. The non-fallers were progressively excluded, by a step of 10%, in order to balance the size of the two groups without excluding fallers. The included subset of each step was selected uniformly at random 12 times, all fallers were included, and the tests run in every iteration. We observe that ts-AUC and MMD have almost equal performance. Decreasing the non-faller population leads to lower chance of distinguishing the two groups. On the other hand, all the two-sample corrections present significantly lower performance.

Regarding Fig 6, that shows the important role of the size proportion among the groups, the performance of ts-AUC, MMD, and multiple testing were comparable to those from Fig 5 (uniform decrease of the population size). However, ts-AUC and MMD exhibit a less abrupt decrease of performance. On the other hand, the gradual balancing of the sizes of the two groups, through the exclusion of non-fallers, seems to have a minor effect on the univariate MWW testing.

## Discussion

The objective of this study was to introduce an easy, interpretable, and intuitive multivariate two-sample testing strategy. The particular interest of this study was to highlight the beneficial effect that this approach can have in clinical research, and particularly in the research of postural control in PS patiens. Using the proposed statistical testing approach, it was shown that: a) Different profiles between fallers and non-fallers were observed only for Eyes-Open protocol; b) The fall-prone PS patients have significantly different statokinesigram profile during quiet standing from those who are non-fallers, contrary to the classic multiple testing approach which did not agree with such a result; c) The novel multivariate two-sample testing approach (ts-AUC) showed equal performance with the state-of-the-art Maximum Mean Discrepancy (MMD) test, with the additional element of providing feature importance assessment without further analysis. d) The VelocityY, VarianceY, AccelerationY, EllArea (Confidence Ellipse area), and MaxX, appeared to be the most important features for distinguishing fallers and non-fallers.

One of the main results of this article is that the proposed multivariate two-sample test, the ts-AUC, and the standard statistics (usually used in clinical studies), when both applied to the dataset of PS patients lead to contradictory conclusions. The multivariate approach found fallers’ and non-fallers’ statokinesigram characteristics significantly different, while traditional statistics did not confirm this result. In line with previous works [6, 8], the applied *p*-value correction strategies are found to be more strict in controlling the Type I error, compared to the proposed multivariate alternative.

Researchers can always perform multiple univariate tests and not apply correction strategies (see univariate MWW results in Table 4, Figs 5, and 6), and take the risk of having a false-positive finding. However, when modest evidence is found in relatively small populations after multiple testing, then the aforementioned false-positive probability is significantly high. The level of that risk may be controlled when some criteria are met (see [6]) considering the quality of the study, the quality of the dataset and the clinical strength of pre-set hypotheses. In exploratory studies though, some of the *p*-values around 0.05, whichever side they may lie on, would definitely be considered as “interesting hints”, whereas concluding without thoughtful consideration from such findings should be generally avoided [13]. The multivariate and cross-validated approaches can decrease the aforementioned uncertainty. The proposed ts-AUC test has interesting and convenient properties: it is a test which is easy to implement and interpret, while it can be also applied to other similar multidimensional datasets.

The features included in our analysis have been used by clinical researchers in the past. Most of them were proposed as indicators of balance impairment at least once in the clinical literature (indicative references [2, 29, 30, 35]). We deliberately avoided any feature engineering or transformation process, not only because that goes beyond the scope of this study, but also because we intended to focus particularly on the merits of the newly proposed approach.

Interestingly, only the Eyes-Open acquisition allowed to significantly distinguish fallers from non-fallers in a population of PS patients. This result seems slight contradictory since PS patients exhibit increased dependency on visual sensing [36]. By exploiting the advantage of the ts-AUC test that provides automatically the importance assessment of features, we found that medio-lateral movement played also a role in faller/non-faller separation of PS patients (see Figs 3 and 4). The medio-lateral movement has been reported as the most discriminative element between PS patients and age-matched controls [5] and seems that play a role in distinguishing fallers and non-fallers PS patients. However, the key-difference between fallers and non-fallers was spotted in antero-posterior movement. VelocityY, VarianceY, and AccelerationY, which may carry overlapping information, were found among the most influential features in the fallers/non-fallers separation. The aforementioned result is in line with previous works that reported increased antero-posterior movement of PS patients in quiet-standing conditions with eyes open [37–39]. Although many PS patients with low postural control did not manifest large posturographic areas, the confidence ellipse area (EllArea) was found significantly larger in fallers compared to non-fallers (Fig 4). However, the EllArea value of non-fallers was highly dispersed. Therefore larger fallers cohorts are needed in order to draw safer conclusions. The confidence ellipse area is recommended to be always considered together with antero-posterior features such as variance and velocity, in order to perform more accurate postural control assessment.

The choice of using the OOB observations as cross-validation method has two basic advantages: 1) provides faster results in the AUC maximization process, and 2) allows the final MWW test to be applied once to the whole dataset, which is more intuitive for clinicians. In cases where the population size is sufficiently large and the hypothesis of similar distributions between train and test-sets is not violated, it is expected that more classic methods such train-test split (as originally proposed in [14]) would have given the same result (or even better; OOB prediction error results have been reported as slightly overestimated [40]). However, clinical datasets are usually limited in size and the aforementioned assumption about the same distribution is not always fully guaranteed. In these cases, multiple train-test splits seem more appropriate whereas they would significantly increase the testing process. OOB observations can be seen as an internal multiple train-test split (one per tree) of the RF (each observation’s prediction is predicted by less than *T* trees) but, conveniently, the final two-sample MWW test is applied once to the whole dataset after the validation process.

Another important modification is the addition of unbiased feature importance through random permutation of OOB observations. We believe that this property is a cornerstone of the proposed approach and inline with the current clinicians’ needs. While they need to know if two groups are (or are not) significantly separated, they are also interested to know the most influential features that lead to the reported result. Although the algorithm offers this convenience, we need to note that feature importance should be treated with extra care. The proposed approach tries to minimize the false conclusions concerning the importance of features when redundant features are present. According to [33], some of the collinear features (relevant to the phenomenon) will be in the final selection, and others will not. This issue is still under research and the current ts-AUC framework can integrate better solutions in the future. A general advice to clinicians can be to check for features exhibiting mutual information before the beginning of the testing process.

The features computed by the basic Romberg test have been reported as relatively inconclusive in distinguishing fallers and non-fallers, mainly due to the lack of realistic conditions of fall [21]. The available patients’ dataset, with its relatively ‘marginal’ separation between fallers and non-fallers (see Table 4), can be considered as an ideal dataset in order to check the performance of the newly proposed approach. We consider MMD algorithm as the gold-standard method in terms of separability of the two groups. The fact that ts-AUC shows similar performance to that of MMD is very important, especially if we think that the proposed ts-AUC can also provide additional information about the most influential features without the need of any supplementary (meta-)analysis. Therefore, it would be fare to say that ts-AUC is competitive in terms of performance, while also boosting the interpretability of the result for the convenience of clinicians.

Interestingly, the decrease of the overall population and the gradual balancing between the groups of fallers and non-faller, showed that the proposed test is less conservative than the multiple testing process (with corrections). Exploratory studies, where a hypothesis about the structure of the dataset is not strictly defined in advance, could benefit from such multivariate approaches.

Comparing the results of the two population reduction schemes, i.e. the uniform reduction of the population versus the reduction of non-fallers (the larger group), we observe that all the statistical tests performed slightly worse in the former case. This was an expected result since fallers were only 24 out of the 123 available PS patients, and thus decreasing the size of that group made the fallers heavily underrepresented in the produced subsample.

### Limitations

The first limitation of this study is the lack of sufficient evidence about the reasons behind falls. The basic Romberg test has been reported to be an insufficient protocol to provide such physiological information [21, 41]. Previous studies proposed richer protocols (including multi-tasking or use of foam surfaces [2, 4, 41]) for postural control assessment of fragile individuals such as PS patients. Undoubtedly, such protocols can have beneficial effect to the faller/non-faller classification, as well as to the impairment assessment of patients (visual, vestibular, somatosensor, nervous system). Yet, among the objectives of this work was to show that basic Romberg test does contain fall risk-related information, whose extraction and full exploitation is largely up to the adequacy of the employed statistical analytics.

It is worth noting that there is always some uncertainty in what patients report as their recent fall experience. Participants who were asked about previous falls might confabulate without a conscious intention to deceive (recall bias). Therefore, some of the non-fallers might be mistakenly labeled as non-fallers. Machine learning algorithms are usually robust to the presence of such noise. Besides, in medical studies the sample size is most usually small, as in ours, and it is required to prepare carefully the population to study. Therefore, this kind of noise is usually minor since patients are actually interviewed by medical experts who can identify subjects that could bring uncertainty to the analysis and exclude them from the sample.

In extreme cases of imbalanced datasets with many negative values and few positive ones, other metrics rather than the AUC, such as the precision-recall (PR) curve, the F_1_ score, or the area under the PR curve, could be more appropriate in order to prevent overfitting [42] (AUC still remains robust to imbalanced datasets). We decided to keep the AUC criterion, which is the one initially proposed by [14], in order to fulfill one of our main objectives: to propose the algorithm as understandable, interpretable and easy-to-implement as possible. In return, as it has been already mentioned, we controlled the leaf size (*LS*) and the number of features (*M*) in the optimization procedure, and we applied cross-validation in each resulting case.

The use of Wii Balance Board (WBB) as a force platform during the acquisition protocol, is another mentionable limitation. The reliability of the WBB as a medical examination tool has been previously questioned [43]. Basic reported drawbacks were: a) the modest agreement with laboratory grade force platforms, b) the lower signal to noise ratio in its recording, and c) the irregular sampling rate [44]. We state that we are perfectly aware of the aforementioned limitations. However, Wii Balance Board presents an increasing popularity in posturography studies as a valid tool for assessing standing balance [24, 25]. It is an inexpensive piece of equipment and hence seems ideal for applications that intend to provide a quick and low-cost first scan of individuals with certain possibility of postural control loss. In addition, recent works [25, 27] showed that a careful pre-processing can mitigate some of its aforementioned drawbacks.

## Conclusions

In this paper we showed that using the proposed ts-AUC two-sample test, which is a based on AUC maximization, faller and non-faller patients who suffer from Parkinsonian syndromes (PS) can actually be distinguished by examining posturographic features that are derived following the basic Romberg protocol. This novel approach was also able to reveal the posturographic features that are significantly different between the two groups (more discriminative). We confirmed that a fall-prone PS patient may manifest wider and more abrupt antero-posterior oscillations and larger posturographic areas compared to a non-faller. This separation appeared statistically less detectable when using more traditional approaches such as multiple testing. Interestingly, the above results were observed only in statokinesigrams derived by the Eyes-Open protocol. The results of our study highlighted that new multivariate methods based on machine learning, such as the ts-AUC test, can play an important role in evaluating the usefulness of simple and inexpensive acquisition protocols as well as the extracted posturographic features. We plan to generalize the current framework. Nevertheless, any extension should investigate the statistical metrics that would be theoretically suitable to be used as optimization criteria.

## Appendix

### I. Additional results in simulated datasets

We conducted additional experiments to test and compare the performance of ts-AUC using simulated datasets and we provide it as a supplement to the analysis on the real use-case of we studied in the main text. The figures appearing below compare ts-AUC with MMD and a multiple testing procedure with *p*-value correction.

#### Simulated data

We created datasets by mixing two independent Gaussian groups. For each dataset we pick:

- the population size (*N* = 100 or 200);
- the proportion of the two groups forming the population (50%/50%, 70%/30%, or 90%/10%);
- the number of dimensions (10, 20, or 30) mimicking the amount of variables that a usual clinical study may have;
- 2/3rd of those dimensions had no difference between the two groups by design (generated using exactly the same average and standard deviation).
- the remaining 1/3rd had a progressively increasing difference in their average (x-axis in all figures below).

**Fig 7:**
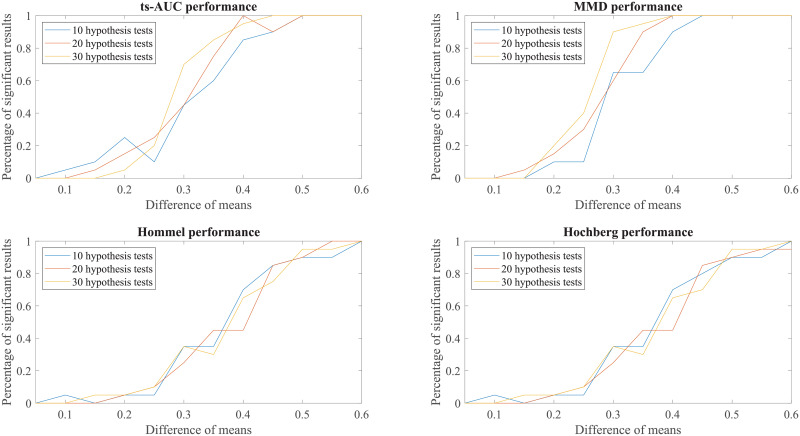
The average performance of two-sample testing approaches in simulated datasets with class balance 50/50, and 10, 20, or 30 features. We observe that ts-AUC and MMD have almost the same performance and always superior to the multiple testing strategies.

**Fig 8:**
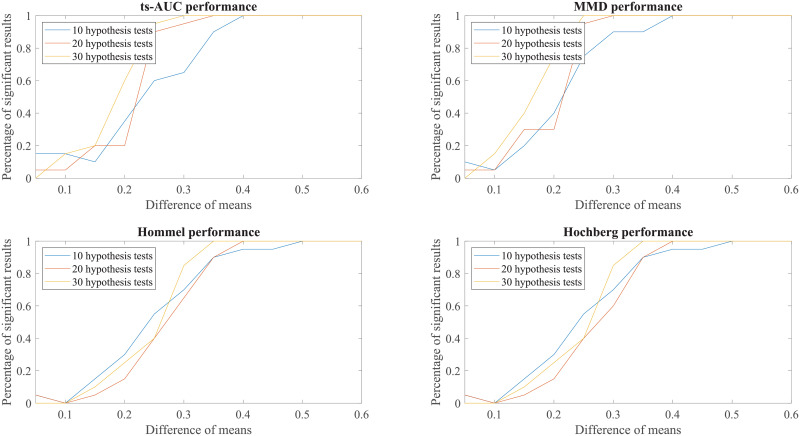
The average performance of two-sample testing approaches in simulated datasets with class balance 100/100, and 10, 20, or 30 features. We observe also that ts-AUC and MMD have almost the same performance and always superior to the multiple testing strategies, especially for the cases of >10 hypothesis tests.

**Fig 9:**
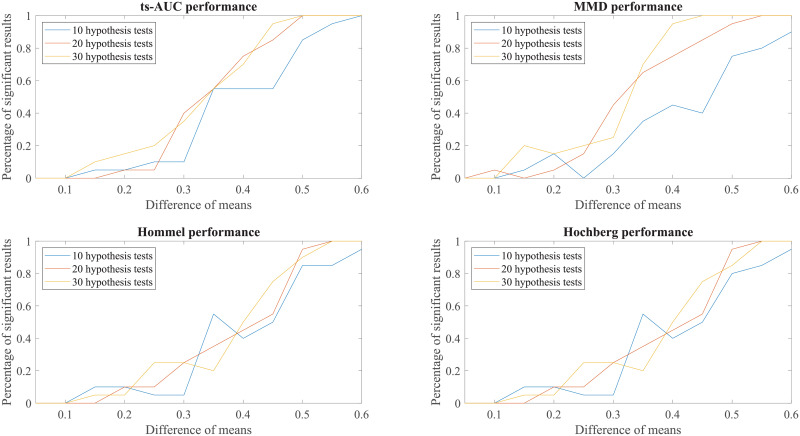
The average performance of two-sample testing approaches in simulated datasets with class balance 70/30, and 10, 20, or 30 features. In this setting, we observe that ts-AUC and MMD have almost the same performance. Introducing class imbalance reduces the chance of distinguishing the two groups mainly due to the low representation of the minor group. The two-sample corrections are affected more and present significantly lower performance than in the balanced case.

**Fig 10:**
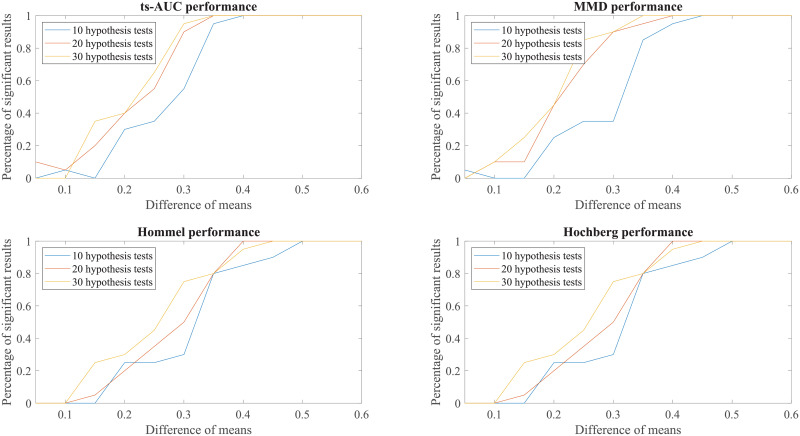
The average performance of two-sample testing approaches in simulated datasets with class balance 140/60, and 10, 20, or 30 features. In this setting, we observe that ts-AUC and MMD have almost the same performance (ts-AUC is slightly better in case of 10 hypothesis test). Some Type I errors might be present in both multivariate tests.

**Fig 11:**
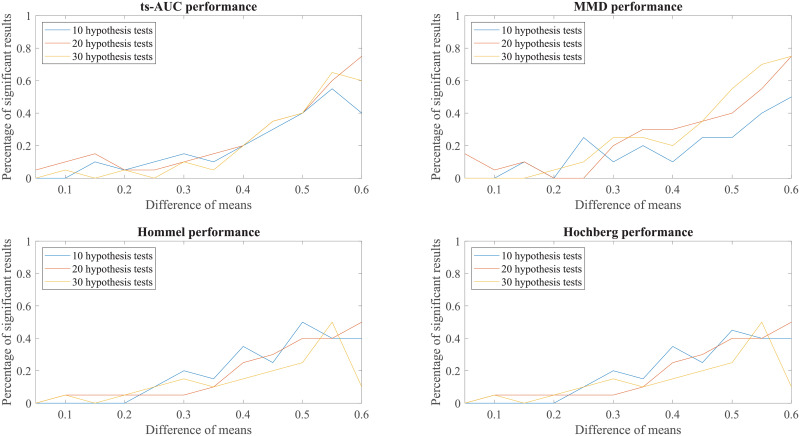
The average performance of two-sample testing approaches in simulated datasets with class balance 90/10. In this setting, we observe that all approaches have almost the same performance. For mean difference >0.5, it seems that the two multivariate approaches, ts-AUC and MMD, begin to have superior performances. However, they also tend to have higher Type I errors. Generally, one of the groups is extremely small (size of 10) for a reliably distinguished distributions at simulation process.

**Fig 12:**
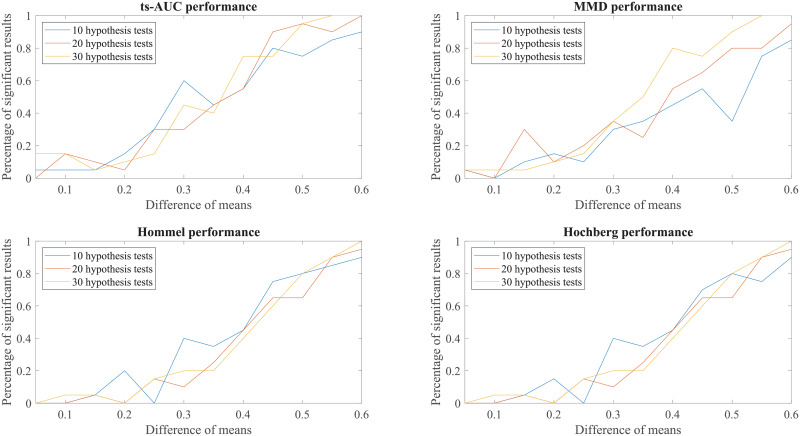
The average performance of two-sample testing approaches in simulated datasets with class balance 180/20. In this setting, we observe that in this special case, still ts-AUC has the best overall performance. We now see more reasonable results due to the fact that the minority group (now size of 20) can marginally have a reliably distinguished distributions at simulation process. However, TYPE I errors are still present.

We run the test 20 times per each combination case. We compared the performance of ts-AUC, MMD, and multiple testing with *p*-value correction (We only mention Hommel and Hochberg for lisibility reasons as well as due to their power superiority compared to the others), keeping the percentage of significant results that each test acquired (y-axis). In all generated cases of non-extreme proportions (50/50, 70/30) between groups’ sizes (Figs 7, 8, 9 and 10), ts-AUC and MMD present similar behavior, and they were always superior to multiple testing approaches in detecting the difference between the two groups. In cases of highly imbalanced groups (see Figs 11 and 12), there is no clear superiority of any method; all methods have increased Type I errors since the generation of the minority group is not reliable.

### II. Feature importance and population

We created two independent Gaussian groups of:

- various total populations (*N* = 50, 100, 150, 200);
- 50%/50% balance between groups;
- 30 dimensions (features);
- three quarters of those features (no. 1-22) had no difference between the two groups by design (all generated using exactly the same average and standard deviation—N(0, 1));
- the remaining one quarter (no. 23-30) were generated by N(0.9, 1);
- no colinearities between features.

**Fig 13:**
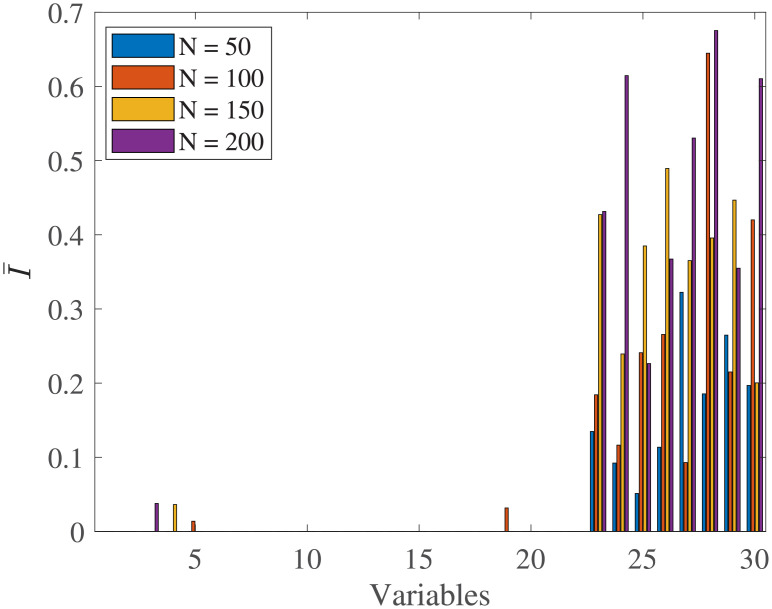
Feature importance relative to the examined overall population size. Features 23-30 are by design significantly different between the two generated groups. We observe that ts-AUC detects effectively the important elements in all populations.

By design, the features 23-30 are significantly different between the two generated groups. We performed 10 runs of the algorithm for every population. Indeed, the feature importance element of the algorithm performed effectively and found as more important the features that by design were more different between the two groups (see Fig 13). The proposed algorithms almost always selected as important elements only those which had by default significant difference between the groups. More details about the limitations of the current feature importance algorithm can be found in the Limitations part at the end of the Discussion section.
